# An evaluation of prospective motion correction (PMC) for high resolution quantitative MRI

**DOI:** 10.3389/fnins.2015.00097

**Published:** 2015-03-25

**Authors:** Martina F. Callaghan, Oliver Josephs, Michael Herbst, Maxim Zaitsev, Nick Todd, Nikolaus Weiskopf

**Affiliations:** ^1^Wellcome Trust Centre for Neuroimaging, UCL Institute of Neurology, University College LondonLondon, UK; ^2^Department of Radiology, University Medical Centre FreiburgFreiburg, Germany; ^3^Department of Medicine, John A. Burns School of MedicineHawaii, HI, USA

**Keywords:** prospective motion correction (PMC), relaxometry, quantitative, multi-parameter mapping, MPM

## Abstract

Quantitative imaging aims to provide *in vivo* neuroimaging biomarkers with high research and diagnostic value that are sensitive to underlying tissue microstructure. In order to use these data to examine intra-cortical differences or to define boundaries between different myelo-architectural areas, high resolution data are required. The quality of such measurements is degraded in the presence of motion hindering insight into brain microstructure. Correction schemes are therefore vital for high resolution, whole brain coverage approaches that have long acquisition times and greater sensitivity to motion. Here we evaluate the use of prospective motion correction (PMC) via an optical tracking system to counter intra-scan motion in a high resolution (800 μm isotropic) multi-parameter mapping (MPM) protocol. Data were acquired on six volunteers using a 2 × 2 factorial design permuting the following conditions: PMC on/off and motion/no motion. In the presence of head motion, PMC-based motion correction considerably improved the quality of the maps as reflected by fewer visible artifacts and improved consistency. The precision of the maps, parameterized through the coefficient of variation in cortical sub-regions, showed improvements of 11–25% in the presence of deliberate head motion. Importantly, in the absence of motion the PMC system did not introduce extraneous artifacts into the quantitative maps. The PMC system based on optical tracking offers a robust approach to minimizing motion artifacts in quantitative anatomical imaging without extending scan times. Such a robust motion correction scheme is crucial in order to achieve the ultra-high resolution required of quantitative imaging for cutting edge *in vivo* histology applications.

## Introduction

Currently, biologically relevant measures of the myelo- and cyto-architecture of the human brain are only available post mortem via histological analysis. As part of the push toward *in vivo* histology, quantitative imaging techniques aim to derive such measures directly from *in vivo* MRI data. A key requirement for such endeavors is ultra-high resolution on the order of hundreds of microns and higher. Recently, the multi-parameter mapping (MPM) approach (Weiskopf et al., 2013) has facilitated *in vivo* mapping with 800 μm isotropic resolution and whole brain coverage allowing for *in vivo* parcellation of the cortex and assessment of structure-function relationships using maps of the longitudinal magnetization relaxation rate (*R*_1_ = 1/*T*_1_) as a surrogate myelin marker (Dick et al., 2012; Lutti et al., 2013; Sereno et al., 2013). Even with the use of accelerating techniques such as parallel imaging, high resolution data sets such as the ones used in these studies inevitably result in long acquisition times and increased sensitivity to motion, particularly as cohorts are extended beyond highly compliant volunteers and into clinical populations. When clinical populations are examined, there is the additional risk that motion artifact can hinder clinical diagnosis by masking or mimicking pathology in the quantitative data (Nöth et al., 2014).

Although inter-volume motion can be compensated to a considerable degree using retrospective rigid body realignment (Friston et al., 1995; Kochunov et al., 2006) the issue of intra-volume motion is far more problematic for 3D acquisitions typical of quantitative anatomical imaging and can render valuable data unusable. A number of approaches have been developed to address intra-volume motion and can be split into two broad categories: retrospective motion correction (RMC) techniques that are applied via post-processing of the acquired data and prospective motion correction (PMC) techniques that monitor and correct for motion at the time of acquisition. RMC approaches based on auto-focusing (Atkinson et al., 1997a,b, 1999; Batchelor et al., 2005; Cheng et al., 2012) have the potential to greatly improve image quality but they also have a number of drawbacks. Estimating the motion trajectory from large raw k-space data sets requires significant computational effort. In addition, they do not compensate for spin history effects nor do they provide an indication of final image quality at the time of data acquisition. Therefore, if residual artifact remains after processing, most notably due to violation of the Nyquist sampling condition when large head rotations occur, rescanning is no longer a possibility. If additional information is used to facilitate RMC, e.g., navigator data or additional scans, there will be an associated acquisition time penalty (Fu and Wang, 1995; Welch et al., 2002; Magerkurth et al., 2011; Cheng et al., 2012; Nöth et al., 2014). This time penalty is also a drawback for PMC approaches that estimate volunteer motion via the MR signal (Welch et al., 2002; Van der Kouwe et al., 2006; White et al., 2010). This time penalty is particularly problematic for rapid imaging sequences, such as Fast Low Angle Shot (FLASH, Haase et al., 1986), that have little or no dead time. An alternative is to use external optical tracking to estimate volunteer motion. This approach to PMC has great promise for flexible and effective motion correction without increasing scan durations (Zaitsev et al., 2006; Maclaren et al., 2012, 2013).

In this study, we assess the performance of such a PMC system (KinetiCor, HI, USA) for use with high resolution quantitative relaxometry mapping. The assessment of PMC techniques is challenging since, unlike their retrospective counterparts, prospective techniques do not produce images with and without motion artifact from the same underlying data. In an effort to gather sufficient baseline data for this study, we used a two factor design. Each constituent volume used to create the quantitative maps was acquired under the four possible conditions permuting *motion* or *no motion* and *PMC on* or *PMC off*. This study assesses the ability of the PMC system to counter both deliberate, bulk head motion, and minimal motion in the range of cardio-respiratory driven physiological motion.

## Materials and methods

### Prospective motion correction

The PMC system employed consists of an optical camera mounted inside the bore of the scanner which tracks the motion of a passive Moire phase marker at a frame rate of 80 Hz. Gratings and patterns on the marker allow the three translational and three rotational degrees of freedom to be measured with precision on the order of tens of microns for the translations and fractions of degrees for the rotations (Maclaren et al., 2012). The tracking information that is logged by the PMC system gives the three translation and three rotation measurements of the marker relative to its center. The rotational precision can have varying impact depending on lighting conditions. In the worst case, assuming a rotational radius of 300 mm (the distance between the marker and the farthest brain location), the precision of 0.01 degrees that has been reported for this system (Maclaren et al., 2012) would produce a translation of order 50 μm at the periphery of the brain.

Information detailing the position and orientation of the marker is sent to the scanner host computer, without any smoothing or filtering. These data are then transformed from camera to scanner coordinates using a pre-calibrated transformation matrix and used to dynamically update the imaging field-of-view (FOV) such that it tracks the movement of the marker, which is assumed to be directly coupled to the movement of the brain (Zaitsev et al., 2006). The imaging gradients, RF frequency and phase are updated in this manner each TR. Motion traces describing the translations and rotations of the marker in scanner coordinates relative to its center are logged for each acquisition.

Since the movement of the marker needs to be as closely coupled to the movement of the brain as possible, bespoke mouth pieces were made for each participant prior to the first scanning session. The mouth pieces consisted of mini bite-bars that were molded to the participant's upper front teeth using a medical grade hydroplastic (TAK Systems, MA, USA). Once securely molded, the mouth pieces remained in place without the participant needing to bite down on them. The marker was then attached to the mouth piece via a lightweight mounting system made from plastic Meccano (www.meccano.com). This mounting system was designed with two pivot points to facilitate flexible positioning of the marker within the field of view of the tracking camera.

### Participants and data acquisition strategy

Six healthy volunteers (5 male; aged 34±7 years) were scanned on a 3T whole body MR system (Magnetom TIM Trio, Siemens Healthcare, Erlangen, Germany) equipped with a standard 32 channel head coil for receive and radiofrequency (RF) body coil for transmission. The study was approved by the local ethics committee and informed written consent was obtained prior to scanning. Data were acquired following a 2 × 2-factorial design permuting the following factors: *motion* and *no motion*; *PMC on* and *PMC off*. This led to lengthy exam durations per volunteer and so the data acquisition was split across two scanning sessions: one for the *motion* condition, the other for the *no motion* condition. Each session lasted approximately 1 h. During the *no motion* session, participants were asked to remain as still as possible. During the *motion* session participants were allowed to move freely within the confines of the tight-fitting local receive coil during the FLASH acquisitions. In all cases the volunteers were blind to whether or not the PMC was on or off, the order of which was randomized.

### Quantitative multi-parameter mapping

Rapid calibration data were acquired at the outset of each session to correct for inhomogeneities in the RF transmit field (Lutti et al., 2010, 2012). These were followed by acquisition of spoiled multi-echo 3D fast low angle shot (FLASH) acquisitions with predominantly proton density (PD), *T*_1_ or MT weighting according to the MPM protocol (Weiskopf et al., 2013). The flip angle was 6^0^ for the PD- and MT-weighted volumes and 21^0^ for the *T*_1_ weighted acquisition. MT-weighting was achieved through the application of a Gaussian RF pulse 2 kHz off resonance with 4 ms duration and a nominal flip angle of 220°. The data were acquired with whole-brain coverage at an isotropic resolution of 800 μm using a FoV of 256 mm head-foot, 224 mm anterior-posterior (AP), and 166 mm right-left (RL). Gradient echoes were acquired with alternating readout gradient polarity at eight equidistant echo times ranging from 2.34 to 18.44 ms in steps of 2.30 ms using a readout bandwidth of 488 Hz/pixel. Only six echoes were acquired for the MT-weighted acquisition in order to maintain a repetition time (TR) of 25 ms for all FLASH volumes. To accelerate the data acquisition, partially parallel imaging using the GRAPPA algorithm was employed in each phase-encoded direction (AP and RL) with forty reference lines and a speed up factor of two. Each FLASH volume was acquired twice with the factor of *PMC on* and *PMC off* randomly ordered.

Quantitative maps were calculated for each condition using bespoke Matlab tools (The Mathworks Inc., Natick, MA, USA) within the SPM12 framework (Ashburner, 2012; Wellcome Trust Centre for Neuroimaging, London). All data were co-registered to address inter-scan motion. Maps of R^*^_2_ were estimated from the gradient echoes from all contrasts using the ordinary least squares ESTATICS approach (Weiskopf et al., 2014). The image data for each acquired weighting (PDw, T1w, MTw) were then averaged over the first six echoes to increase the signal-to-noise ratio (SNR) (Helms and Dechent, 2009). The three resulting volumes were used to calculate MT, *R*_1_ and effective proton density (PD^*^) maps as previously described (Helms et al., 2008a; Weiskopf et al., 2013). The MT map depicts the percentage loss of signal (MT saturation) that results from the application of the off-resonance MT pre-pulse and the dynamics of the magnetization transfer (Helms et al., 2008b). The PD^*^ maps were calculated from the averaged multi-echo FLASH data, which has an effective TE of 8.1 ms and are referred to as effective proton density because there was no correction for T^*^_2_ signal decay.

### Evaluation of PMC impact

To assess the overall impact of the PMC system, histograms of quantitative values across brain voxels were calculated for each quantitative map and each condition. For more detailed intra-cortical analysis, the automated anatomical labeling (AAL) atlas (Tzourio-Mazoyer et al., 2002) was used to define regions-of-interest (ROIs). The unified segmentation approach (Ashburner and Friston, 2005) as implemented in SPM12 was used to create constituent tissue class probabilities and an inverse deformation field from the MT maps for each volunteer and each condition. The MT maps were used for segmentation because of their superior contrast within deep gray matter (GM) structures (Helms et al., 2009). The AAL labels (116 in total) were then transformed from MNI space to the individual's native space using the subject-specific inverse deformation fields. To reduce partial volume effects with cerebrospinal fluid (CSF) and white matter (WM), a liberal threshold of 80% applied to the GM probability determined which voxels were included in the analysis. For each ROI in the atlas, a coefficient of variation (CoV) was calculated separately for each of the quantitative parameters (*R*_1_, R^*^_2_, MT, PD^*^) as the standard deviation of the constituent voxels divided by their mean. Note that this noise measure assumed that the quantitative measures do not vary within a specific brain area. As a global measure, the median CoV across these ROIs was also calculated for each volunteer and each quantitative map. Given the small cohort (six participants) used in this study and the fact that the global CoV measures may not be normally distributed, non-parametric Wilcoxon signed rank tests were used to test for significant pair-wise differences related to motion and the use of PMC at the between-subject level. One-tailed tests were used to determine (a) if motion significantly increased the CoV in the absence of the PMC system and (b) if the PMC system reduced the CoV in the presence of deliberate head motion. A two-tailed test was used to assess the impact of the PMC system in the case of no deliberate head motion. The threshold for statistical significance was set to 0.05/3 = 0.0167 to correct for multiple comparisons. These tests were carried out on each quantitative map independently.

## Results

**Figure 1:**
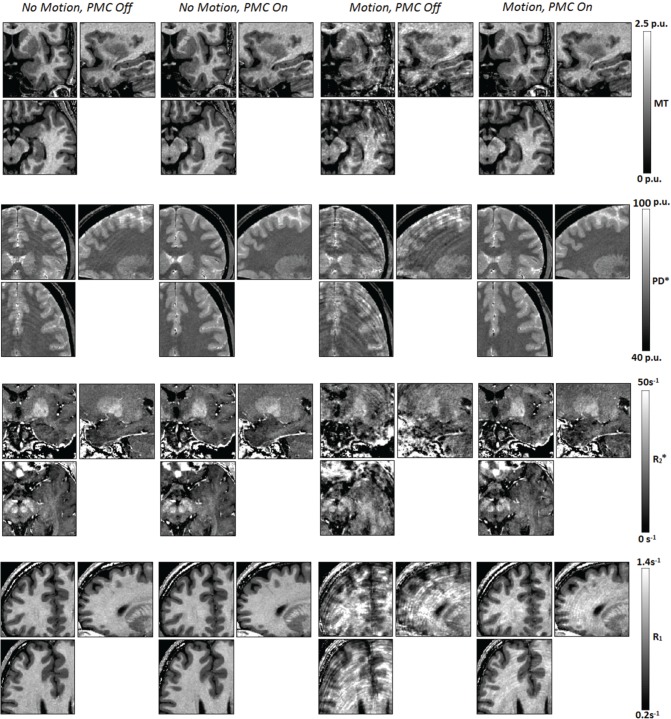
**Quantitative maps across conditions focussing on spatial regions in which features are obscured due to volunteer motion during the acquisition of the constituent weighted volumes**. The manifestation of the motion in the maps was dependent on the coherence of the artifact in the component volumes. Each set of a particular parameter map was acquired from a different volunteer within the cohort (Volunteers 1, 3, 4, and 6 are shown, respectively in descending rows).

**Figure 2:**
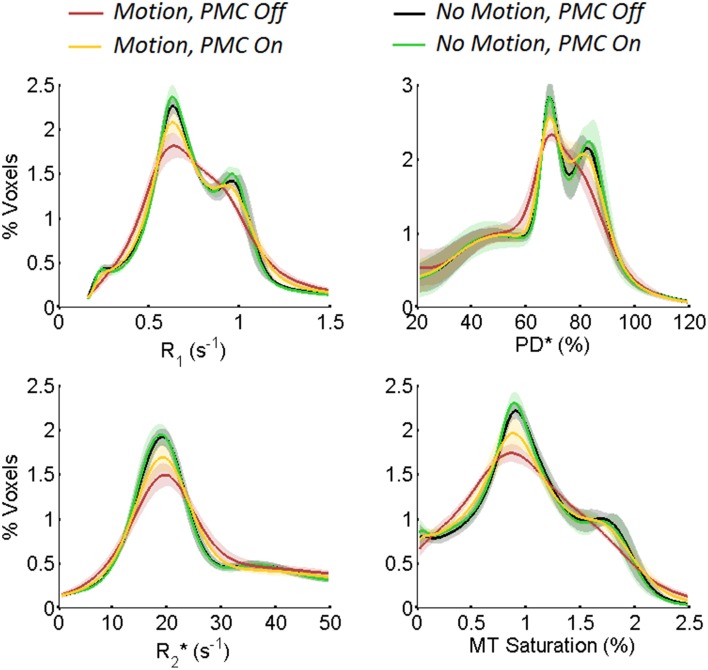
Histograms of quantitative MR parameters across brain voxels, namely longitudinal relaxation rate (R_1_), effective proton density (PD^*^), effective transverse relaxation rate (R^*^_2_), and MT saturation. The shaded area of each curve depicts one standard deviation across all six volunteers. Motion leads to broadening of the peaks, which is considerably reduced by use of the PMC system, recovering the distinct gray and white matter peaks.

Figure 1 shows exemplar quantitative maps across the four experimental conditions, zoomed in to highlight the effects of motion on the quantitative maps. To provide a comprehensive illustration, each set of a given parameter map was derived from a different volunteer. Figure 2 shows histograms of the quantitative parameters. The shaded area of each curve demarks one standard deviation across volunteers. In the presence of motion, the width of the histograms broadened and the gray and white matter peaks converged (red). Under comparable motion conditions, the PMC system sharpened the histogram peaks (yellow), such that they approached the level of the *no motion, PMC Off* case (black). The histograms were further sharpened when PMC was used with no deliberate motion (green). These characteristics were common to all maps and volunteers.

**Figure 3:**
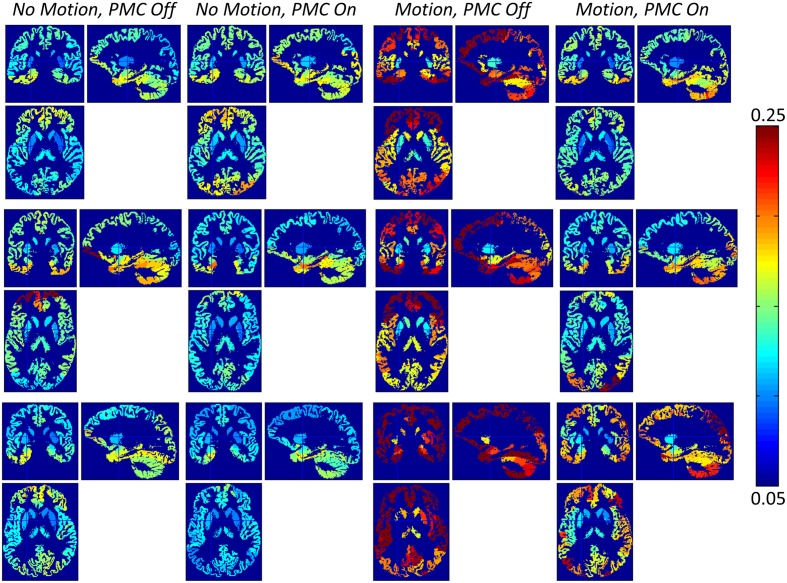
**Spatial maps of the coefficient of variation of *R*_1_ for three volunteers**. Motion increased the CoV, but to a lesser extent when using the PMC system. In the absence of intentional motion, the CoV was increased by the PMC system for volunteer 1 (top row) but reduced for all other volunteers, e.g., volunteer 3 (middle row) and volunteer 4. Volunteer 4 (bottom row) moved very rapidly in the *motion* condition limiting the improvement gained by the PMC system. The rapid motion resulted in poor segmentation for the *motion, PMC off* condition.

**Figure 4:**
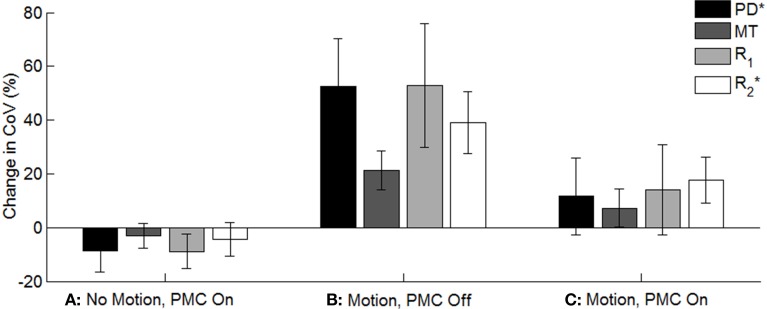
**Median change in the global CoV measure relative to the standard condition of *no motion, PMC off* across all volunteers for each of the other conditions investigated and for all quantitative maps**. The error bars denote the inter-quartile range across all volunteers.

Figure 3 shows spatial maps of the CoV of *R*_1_ from three volunteers in AAL-defined regions of interest. Motion greatly increased the CoV (columns three and four), but to a lesser extent when the PMC system was on (column four). The impact of the *motion* and *PMC* factors varied spatially. Within the condition of *motion, PMC off* the CoV was higher anteriorly (column three) reflecting the fact that movement of the head was restricted posteriorly with volunteers in the supine position. Within the condition of *motion, PMC on* the CoV was higher inferiorly (column 4, e.g., in the cerebellum) where the assumption of rigid body motion is less valid (Greitz et al., 1992; Soellinger et al., 2009). Figure 4 summarizes the group differences in the global CoV measure relative to the *no motion, PMC off* condition for each map. In the *motion, PMC off* case the CoV was significantly increased (Figure 4B, *p* = 0.0156 for each map). The increases were 0.042 ± 0.012 (median ± inter-quartile range) for PD^*^, 0.039 ± 0.012 for MT, 0.076 ± 0.039 for *R*_1_ and 0.125 ± 0.039 for R^*^_2_. These changes corresponded to a median CoV increase with respect to the *no motion, PMC off* condition of 52.6, 21.3, 52.7, and 39.1% for PD^*^, MT, *R*_1_ and R^*^_2_, respectively (Figure 4B) showing that motion had the greatest impact on variance levels in the PD^*^ and *R*_1_ maps.

Within the motion condition, i.e., comparing *motion, PMC off* and *motion, PMC on*, the CoV was significantly decreased with the PMC system on (*p* = 0.0156 for each map). The decreases were 0.028 ± 0.007 for PD^*^, 0.025 ± 0.018 for MT, 0.052 ± 0.019 for *R*_1,_ and 0.059 ± 0.033 for R^*^_2_. These changes corresponded to relative reductions in median CoV with respect to the *motion, PMC off* condition of 21.8, 11.3, 25.0, and 12.6% for PD^*^, MT, *R*_1_ and R^*^_2_, respectively. Although the CoV of the *motion, PMC on* case was significantly reduced with respect to the *motion, PMC off* case, the CoV remained higher than the *no motion, PMC off* case (Figure 4C).

When there was no intentional motion, the effect of the PMC system did not reach significance. The CoV was reduced in all maps for five out of the six volunteers. The net changes between *no motion, PMC off* and *no motion, PMC on* across the group were −0.008 ± 0.008 for PD^*^, −0.006 ± 0.008 for MT, −0.013 ± 0.010 for *R*_1_ and −0.013 ± 0.021 for R^*^_2_. This corresponds to a median reduction in CoV with respect to the *no motion, PMC off* condition of 8.8, 3.1, 8.8, and 4.4%, respectively for PD^*^, MT, *R*_1_ and R^*^_2_ (Figure 4A).

## Discussion

Motion artifact can significantly degrade the quality and precision of quantitative parameter maps. The results of the analyses on the cohort of six volunteers that participated in this study show that using a PMC system significantly improves the quality of quantitative MRI parameter maps in most cases, particularly in the presence of motion, where median CoV reductions for the group ranged from 11 to 25% depending on the parameter map, but also under the condition of no deliberate volunteer motion where the CoV can also be reduced.

There are an infinite number of head motion trajectories for which the performance of any motion correction approach could be investigated. In order to assess the general feasibility of the approach volunteers were not restricted to particular trajectories but rather told that they could move freely. In an additional effort to minimize bias between conditions, the order of the PMC on vs. PMC off conditions were randomized and volunteers were blinded to this condition. Volunteers reported adopting strategies of looking around, moving their legs, mimicking patients they had previously scanned or making movements associated with falling asleep and waking up. Clearly it is important that the range of motion be representative of what might be expected during routine scanning, particularly of more difficult cohorts. Summary statistics from the tracking data generated in this study that describe the Euclidean displacement of the marker are presented in Table 1. The metrics for the *no motion* case are on a par with those previously reported for patient groups (Kochunov et al., 2006; Versluis et al., 2010) while the *motion* metrics are far higher. This indicates that the findings of the present study will have broad and general applicability, since they most likely address a worst case scenario.

**Table 1:** **Motion across the FLASH volumes are summarized as mean, standard deviation (SD), and maximum Euclidean displacement of the marker for each volunteer and experimental condition**.

**Volunteer**	**No Motion**		**Motion**	
	**Mean ± SD**	**Max**	**Mean ± SD**	**Max**
1	1.05 ± 0.60	6.86	2.76 ± 1.69	13.80
	1.08 ± 1.05	4.35	3.05 ± 2.25	11.48
2	1.74 ± 0.84	3.43	6.37 ± 5.35	20.87
	1.47 ± 0.42	2.36	6.07 ± 3.45	17.59
3	0.62 ± 0.41	5.80	2.88 ± 2.74	16.45
	0.72 ± 0.42	3.92	2.11 ± 1.80	11.39
4	2.18 ± 1.05	4.84	4.07 ± 2.32	12.47
	1.69 ± 0.98	3.99	3.73 ± 2.03	25.04
5	1.05 ± 0.69	2.97	4.08 ± 3.77	14.55
	0.87 ± 0.49	2.35	3.78 ± 2.81	25.04
6	0.62 ± 0.48	2.29	3.61 ± 2.38	15.28
	0.66 ± 0.45	2.04	3.50 ± 2.18	14.93

*The upper row for each volunteer corresponds to the PMC Off condition, while the lower row corresponds to the PMC On condition. All metrics are in millimeter units*.

Motion-related artifacts can manifest in quantitative maps as the striations typical of motion artifact in conventional weighted volumes. This is most notable in the PD^*^ maps in Figure 1 when PMC is off, both in the *motion* and *no motion* cases. Some robustness to this form of motion artifact is inherent in the creation of the quantitative maps when the motion is not coherent across the constituent weighted volumes. However, the underlying signal intensity remains erroneous leading to focal hypo- and hyper-intense values in the derived maps. This effect is particularly evident in the *R*_1_ and MT maps in Figure 1. We have used the coefficient of variation over sub-regions of the cortex as an assessment metric in order to be sensitive to these irregular values, assuming that the sub-region's tissue composition does not vary across the region. To provide a more global view of the data quality and assess the differentiation between gray and white matter, histograms of the whole brain parameter distribution were used.

Considering the cases when PMC was not used (i.e., *motion, PMC off* vs. *no motion, PMC off*), volunteer motion significantly increased the global coefficient of variation metric within all quantitative maps indicating that this metric captures the effect of motion and is appropriate for assessing the impact of the PMC system. Under the condition of deliberate volunteer motion (i.e., *motion, PMC on* vs. *motion, PMC off*) the global CoV was significantly reduced in all quantitative maps when the PMC system was used. This confirms the PMC system as a robust means of addressing volunteer motion and improving the quality of quantitative MR parameter maps. Uncorrected rapid motion was also seen to introduce artifact that degraded the performance of the segmentation in the *motion, PMC off* condition but was improved by using the PMC system (compare motion conditions in row 3 of Figure 3). The CoV for the *motion, PMC on* case remains higher (see Figure 4, 7–16% for the group) than the *no motion* cases indicating that the PMC system cannot account for all effects of volunteer motion. This is to be expected since the PMC system only corrects for rigid body motion. It cannot correct for additional non-rigid body motion and position-dependent effects, such as the rapidly varying sensitivity profile of the 32 channel receive coil, shim changes and spatially varying gradient performance (for a full review see Maclaren et al., 2013).

**Figure 5:**
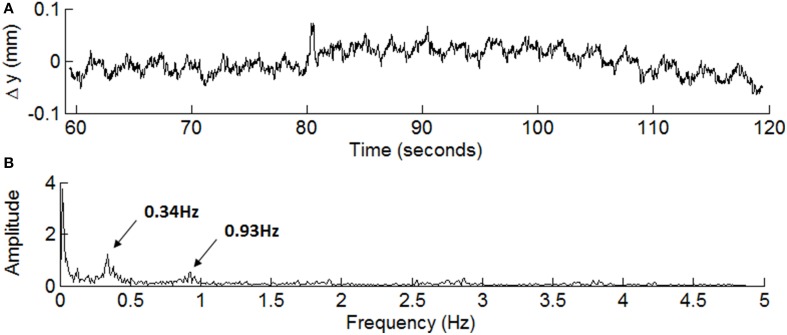
**A 1 min segment of the motion trace depicting (A) translation in the y-direction (posterior–anterior) from volunteer 6 under the no motion condition after linear detrending, along with (B) the extracted power spectrum**. Peaks can be seen in the power spectrum at 0.34 and 0.94 Hz consistent with breathing and cardiac cycles, respectively.

Another important criterion for adopting a PMC system is that it does not in itself degrade the quality of the acquired imaging data. We assessed this via the data from the *no motion* factor, since it would most likely show any additional noise introduced by a PMC system. In this case, the peaks of the group histograms are sharpened when the PMC system is used. Five out of six volunteers showed reduced global CoV in all quantitative maps. Thus, the PMC system may also be used to reduce artifacts even in high quality datasets affected only by minimal motion, suggesting that the correction of artifacts introduced by physiological motion, e.g., due to breathing or the cardiac cycle, may be possible. Figure 5 shows a 1 minute long segment of the y-translation (posterior-anterior direction) motion trace for volunteer 6. The corresponding power spectrum (Figure 5B) identifies peaks in the spectrum at 0.34 Hz, consistent with breathing, and at 0.94 Hz, consistent with the cardiac cycle.

The remaining volunteer showed increased CoV in the *no motion, PMC on* case relative to the *no motion, PMC off* case. This is likely due to inadequate marker fixation. Secure marker fixation is crucial in order to satisfy the assumption of direct coupling between the marker and the brain. Imperfect coupling may not detect genuine brain movement, or worse, may introduce erroneous corrections and therefore inadvertently cause motion artifact. This is particularly problematic for the *no motion* case since the PMC system will only be beneficial if it improves alignment between the scanner and the true volunteer position (Herbst et al., 2014). This is more likely to be the case in the presence of deliberate motion, even with only moderately good coupling. This is evidenced by the fact that there was a clear benefit in using the PMC system for this volunteer in the presence of motion (see MT saturation maps in Figure 1 and CoV in top row of Figure 3). In the planning stage of this study a number of simpler alternatives to the bespoke bite-bar solution were investigated, e.g., affixing the marker to the nasal bone or to MR-compatible glasses. However, these solutions showed higher variability in the tracking data that was not correlated with brain motion. While the mini-bite-bars produce lowest noise in the tracking data, they also have certain drawbacks. Such a device may not be well-tolerated by all volunteers and patient groups and requires significant time, since an extended or additional visit is required, and effort to construct. It is also difficult to evaluate prior to scanning whether or not the coupling efficiency will be sufficient.

The correction performance also depends on the temporal resolution and latency of the PMC system with respect to updating the imaging gradients. In this study, updates were applied every TR, i.e., 25 ms, assuming no motion within the TR. An alternative would be to update every gradient event (e.g., readout, spoilers). However, this would require an order of magnitude increase in the temporal resolution of the system rather than the factor of two that would be available. The latency of the system from tracking a position to updating the scanner is approximately 30 ms. This limits the movement velocities that can be corrected. No filtering or outlier correction was applied to the tracking data. A predictive filtering approach, e.g., using a Kalman filter, might increase robustness both to the latency of the system and to the issue of marker fixation. This will be a focus of future work.

## Conclusions

Intra-scan motion is a significant problem for MRI in general and for high resolution quantitative imaging in particular. PMC provides an effective means of addressing this source of artifact. We have demonstrated considerably improved precision, in the region of 11–24%, in measuring relaxometry, effective proton density and magnetization transfer saturation maps in the presence of motion using this approach. Importantly, we have also shown that, provided there is good coupling between the marker and the brain, the system does not introduce extraneous artifacts in cases where there is no deliberate motion and may additionally correct for microscopic involuntary motion. We anticipate that the use of robust PMC will be key for achieving the ultra-high resolution required of quantitative imaging for *in vivo* histology applications.

### Conflict of interest statement

The Wellcome Trust Centre for Neuroimaging has an institutional research agreement with and receives support by Siemens. The authors declare that the research was conducted in the absence of any commercial or financial relationships that could be construed as a potential conflict of interest.
